# Increase in non-tuberculous mycobacteria isolated from humans in Tuscany, Italy, from 2004 to 2014

**DOI:** 10.1186/s12879-016-1380-y

**Published:** 2016-02-01

**Authors:** Laura Rindi, Carlo Garzelli

**Affiliations:** Dipartimento di Ricerca Traslazionale e delle Nuove Tecnologie in Medicina e Chirurgia, Università di Pisa, Via San Zeno, 35/39, 56127 Pisa, Italy

**Keywords:** Non-tuberculous mycobacteria, Human mycobacteriosis, NTM Epidemiology

## Abstract

**Background:**

In Italy, the prevalence of non-tuberculous mycobacteria (NTM) in human infections is largely unknown. Herein, we report the epidemiology of NTM infections in a region of central Italy, Tuscany, over the last 11 years, and provide a review of the recent literature on NTM isolation rates in different geographic regions.

**Methods:**

The complete collection of NTM strains isolated from a total of 42,055 clinical specimens at the Laboratory of Clinical Mycobacteriology of Pisa University Hospital, Italy, from 1 January 2004 to 31 December 2014 was included.

**Results:**

In our setting, in the period 2004–2014 a total of 147 patients had cultures positive for NTM. The number of NTM isolates increased considerably from five isolates in 2004 to 29 in 2014; a sharp increase occurred in the last 3 years. Overall, 16 NTM species were isolated; the most common were *M. avium, M. intracellulare* and *M. gordonae* detected in respectively in 41.5, 14.3 and 11.6 % of NTM patients. In general, NTM isolates were largely prevalent in people older than 60 (57.8 %); patients aged 1–10 year-old almost exclusively yielded *M. avium* and *M. intracellulare*. Of the 147 NTM clinical isolates, 76.2 % were from respiratory specimens, 10.9 % from lymph nodes, 2.7 % from blood (yielding exclusively *M. avium*), and the remaining 10.2 % from other clinical specimens.

**Conclusions:**

The observed increase in NTM isolation rate in our setting is in keeping with the general increase in NTM infections reported worldwide in the past two decades, although the distribution of the NTM prevalent species differs by geographic region.

## Background

Non-tuberculous mycobacteria (NTM) include all *Mycobacterium* species other than *Mycobacterium tuberculosis* complex and *Mycobacterium leprae*. NTM are a group of over 150 enviromental species, but, due to recent advancement of molecular techniques, novel species are being identified [1]. NTM are generally endowed with low pathogenicity to humans [2], however some species are associated with a variety of human diseases, especially concomitantly to particular risk factors [3]; respiratory tract infections are the most frequent, followed by lymphadenitis in children, disseminated infections in severely immunocompromised patients and skin infections [4]. Although significant differences in geographic distribution of NTM have been observed [5], species belonging to *Mycobacterium avium* complex (MAC), particularly *M. avium* and *M. intracellulare*, are the most frequently reported; other important human NTM pathogens include slowly growing mycobacteria, usually community-acquired from environmental or animal sources, such as *Mycobacterium kansasii*, *Mycobacterium xenopi*, *Mycobacterium malmoense,* and rapidly growing mycobacteria typically hospital-acquired, such as *Mycobacterium abscessus*, *Mycobacterium chelonae* and *Mycobacterium fortuitum*. A considerable increase in both incidence and prevalence of NTM infections has been observed worldwide in the past two decades, not only for the development of new and more sensitive molecular methods for NTM identification, but also for a real increase in NTM disease cases [6]. Several European countries have published studies about NTM epidemiology showing an increase in NTM isolated from human clinical samples [7–9], however NTM epidemiology in Italy is largely unknown. The aim of the present survey is to provide an overview of the epidemiology and recent trend of NTM infections in a region of central Italy, Tuscany, over the last 11 years.

## Methods

### Clinical isolates

The survey include the complete collection of 147 NTM strains, isolated from the same number of patients, from a total of 42,055 clinical specimens at the Laboratory of Clinical Mycobacteriology of Pisa University Hospital, Italy, during an 11-year study period from 1 January 2004 to 31 December 2014. In the case of multiple consecutive positive cultures from the same patient, only the first isolate was included in the present study. All strains were isolated by using the BACTEC MGIT960 liquid culture system (Becton Dickinson, USA) and were identified by molecular probes (InnoLipa [Innogenetics, Belgium] and/or Genotype CM/AS [Hain Lifescience, Germany]) and by a multiplex PCR designed to discriminate MAC organisms [10]. Patients’ clinical information, including gender, age and site of infection, was obtained from clinical records; distinction between community-versus hospital-acquired infections was not possible.

Research ethics approval was not necessary for retrospective studies in our Institution; informed consent was not required as the data were analyzed anonymously.

## Results and discussion

Between January 2004 and December 2014, a total of 42,055 clinical specimens collected from approximately 15,000 patients with suspected mycobacterial infection were tested; a total of 595 patients had cultures positive for mycobacteria; *M. tuberculosis* complex and NTM species were isolated from 448 (75.3 %) and 147 (24.7 %) patients, respectively. A total of 16 NTM species were isolated (Table 1), the most common belonging to the MAC (*n* = 82, 55.8 %); in particular, *M. avium* subsp. *hominissuis* was detected in 61 (41.5 %) and *M. intracellulare* in 21 (14.3 %) patients. *M. gordonae* was the third prevalent species (*n* = 17, 11.6 %), followed by *M. xen*opi (*n* = 14, 9.5 %), *M. fortuitum* (*n* = 10, 6.8 %), and *M. kansasii* (*n* = 7, 4.8 %). In general, the species distribution of NTM isolated in our setting was close to that reported earlier in an inventory study of NTM in the European Union, which included Italy [7]. Similarly to other studies [9, 11], the NTM infections were not associated with gender, although *M. intracellulare* and *M. kansasii* appeared to be more common in men (16 males *vs* 5 females, *P* = 0.002 for *M. intracellulare;* 6 males *vs* 1 females, *P* = 0.003 for *M. kansasii*, by χ2 analysis). In general, NTM isolates were largely prevalent in people older than 60 (*n* = 85, 57.8 %)¸ patients aged 1–10 year-old almost exclusively yielded *M. avium* and *M. intracellulare* (11 out of 12, 91.7 % in total), the most commonly encountered species in mycobacterial lymphadenitis in children [12]. Of the 147 NTM clinical strains, 112 (76.2 %) were isolated from respiratory tract specimens (sputum and bronchoalveolar lavage), 4 (2.7 %) from blood, 16 (10.9 %) from lymph nodes, and the remaining 15 isolates (10.2 %) from other specimens, including urine, stool, skin, gastric lavage and other body fluid. In particular, all the blood specimens yielded *M. avium*, which was also the prevalent species isolated from adenitis episodes; *M. xenopi* and *M. kansasii* were isolated only from pulmonary specimens; *M. marinum* was isolated exclusively from skin samples. These results reflect the ability of the mycobacterial species to infect and localize in different body sites [3].

**Table 1:** Species, patients’ age and site of infection of NTM isolated at the Clinical Mycobacteriology Laboratory of Pisa University Hospital during years 2004–2014

NTM species	No. of isolates (% of total isolates)	No. of isolates (% of species isolates)												
		Patient age range									Site of isolation			
		1–10	11–20	21–30	31–40	41–50	51–60	61–70	71–80	>80	Respiratory tract	Blood	Lymph node	Other^a^
*M. avium*	61 (41.5)	8 (13.1)	2 (3.3)	1 (1.6)	–	8 (13.1)	7 (11.5)	9 (14.8)	20 (32.8)	6 (9.8)	40 (65.6)	4 (6.6)	11 (18.0)	6 (9.8)
*M. intracellulare*	21 (14.3)	3 (14.3)	–	–	2 (9.5)	–	2 (9.5)	5 (23.8)	6 (28.6)	3 (14.3)	17 (81.0)	–	4 (19.0)	–
*M. gordonae*	17 (11.6)	–	–	–	3 (17.6)	4 (23.5)	2 (11.8)	3 (17.6)	4 (23.5)	1 (5.9)	14 (82.4)	–	–	3 (17.6)
*M. xenopi*	14 (9.5)	–	–	–	5 (35.7)	2 (14.3)	2 (14.3)	4 (28.6)	–	1 (7.1)	14 (100.0)	–	–	–
*M. fortuitum*	10 (6.8)	–	–	–	–	1 (10.0)	2 (20.0)	3 (30.0)	4 (40.0)	–	7 (70.0)	–	–	3 (30.0)
*M. kansasii*	7 (4.8)	–	–	1 (14.3)	–	–	1 (14.3)	4 (57.1)	1 (14.3)	–	7 (100.0)	–	–	–
*M. celatum*	3 (2.0)	1 (33.3)	–	–	–	–	–	1 (33.3)	1 (33.3)	–	2 (66.6)	–	1 (33.3)	–
*M. abscessus*	2 (1.4)	–	–	–	–	–	–	–	1 (50.0)	1 (50.0)	1 (50.0)	–	–	1 (50.0)
*M. chelonae*	2 (1.4)	–	–	–	1 (50.0)	1 (50.0)	–	–	–	–	2 (100.0)	–	–	–
*M. marinum*	2 (1.4)	–	–	–	–	2 (100.0)	–	–	–	–	–	–	–	2 (100.0)
*M. lentiflavum*	2 (1.4)	–	–	–	–	–	–	2 (100.0)	–	–	2 (100.0)	–	–	–
*M. simiae*	2 (1.4)	–	–	–	–	–	1 (50.0)	–	1 (50.0)	–	2 (100.0)	–	–	–
*M. scrofulaceum*	1 (0.7)	–	–	–	–	–	––	–	1 (100.0)	–	1 (100.0)	–	–	–
*M. triplex*	1 (0.7)	–	–	–	–	–	–	–	–	1 (100.0)	1 (100.0)	–	–	–
*M. phocaicum*	1 (0.7)	–	–	–	–	–	–	–	–	1 (100.0)	1 (100.0)	–	–	–
*M. bolletii*	1 (0.7)	–	–	–	–	–	–	–	1 (100.0)	–	1 (100.0)	–	–	–
Total	147 (100.0)	12 (8.2)	2 (1.4)	2 (1.4)	11 (7.5)	18 (12.2)	17 (11.6)	31 (21.1)	40 (27.2)	14 (9.5)	112 (76.2)	4 (2.7)	16 (10.9)	15 (10.2)

^a^include isolates from urine, stool, skin, gastric lavage and other body fluids

**Fig. 1:**
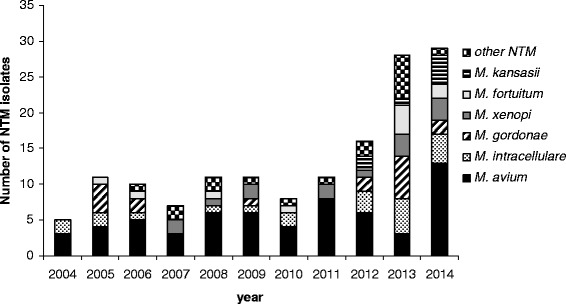
NTM isolated at the Clinical Mycobacteriology Laboratory of Pisa University Hospital during years 2004–2014. Only the six prevalent species, out of the 16 isolated during the survey period, are individually reported with different column fillings; the other NTM species (*M. celatum, M. abscessus, M. chelonae, M. marinum, M. lentiflavum, M. simiae, M. scrofulaceum, M. triplex, M. phocaicum* and *M. bolletii*) are grouped together (see figure legend at the right side)

The distribution over time of the NTM isolates is reported in Fig. 1. As shown, the number of NTM isolates increased considerably from five isolates in 2004 to 29 in 2014; a sharp increase occurred in the last 3 years. In 2014, in particular, *M. avium* and *M. intracellulare* were the prevalent isolates representing 58.6 % of total NTM isolates; the increase in MAC isolates occurred mostly in people aged over 60 with pulmonary infections, as also reported by others [9, 13, 14]. Notably, *M. kansasii*, a pulmonary pathogen not reported before 2012 in our setting, was repeatedly isolated in the last 3 years, representing 9.6 % of total NTM isolates; *M. gordonae*, which is considered a non-pathogenic enviromental contaminant, was the third most frequently isolated species.

On the whole, the increase in isolation rate of NTM in our setting in recent years is in keeping with several reports all over the world [7–9, 11, 15–19]. However, it is worthy to note that the distribution of the species of NTM isolated from clinical specimens differs markedly by geographic region. Table 2 summarizes a selection of the recent literature reporting NTM isolation rates in different settings and geographic regions. In Europe, *M. avium* and *M. intracellulare* were the most common species, reaching prevalence rates approximately as high as 40–60 % in Italy, United Kingdom and the Netherlands. Interestingly, in the United Kingdom, although *M. avium-intracellulare*, *M. malmoense* and *M. kansasii* were the prevalent species, the rise in NTM isolates was mostly due to *M. gordonae* generally isolated from pulmonary specimens in patients over 60 year-old [9, 13]; similarly, in a study from Croatia, the highest increase in NTM isolation frequency was attributed to *M. gordonae* and *M. fortuitum* [11]; also studies from the Netherlands and Athens (Greece) showed high rates of *M. gordonae* isolates [14, 20]. In the United States the prevalent NTM species were *M. avium* complex, *M. gordonae* and *M. abscessus* [21, 22], while in Eastern Asia the most frequent were *M. avium* complex, *M. kansasii* and *M. abscessus* and *M. fortuitum* [23–25].

**Table 2:** Recent studies on isolation rates of NTM from human clinical specimens in different settings

Setting	Years	Prevalent NTM species	% of total isolates	Trend	Reference
Tuscany (Italy)	2004–2014	*M. avium*	41.5	Increase	This study
		*M. intracellulare*	14.3		
		*M. gordonae*	11.6		
England, Wales and Northern Ireland	1995–2006	*M. avium-intracellulare*	42.9	Increase	9
		*M. malmoense*	13.7		
		*M. kansasii*	12.5		
Scotland	2000–2010	*M. avium* complex	48.1	No clear trend	13
		*M. malmoense*	17.7		
		*M. abscessus*	9.8		
The Netherlands	2000–2006	*M. avium* complex	39.0	Increase	14
		*M. gordonae*	14.1		
		*M. kansasii*	7.4		
Croatia	2006–2010	*M. gordonae*	42.9	Increase	11
		*M. xenopi*	15.5		
		*M. fortuitum*	11.5		
Athens (Greece)	2007–2013	*M. gordonae*	13.9	No trend	20
		*M. avium*	13.1		
		*M. fortuitum*	12.2		
Virginia (USA)	2001–2009	*M. avium* complex	40.9	Increase	22
		*M. gordonae*	28.7		
		*M. abscessus*	4.5		
Oregon (USA)	2007–2012	*M. avium-intracellulare*	86	Increase	21
		*M. chelonae/abscessus*	6		
Shangai (China)	2008–2012	*M. kansasii*	45.0	Increase	23
		*M. intracellulare*	20.8		
		*M. chelonae/abscessus*	14.9		
Cheonan (Korea)	2005–2011	*M. intracellulare*	51.3	Increase	24
		*M. avium*	14.7		
		*M. kansasii*	7.8		
South Korea	2001–2011	*M. avium* complex	53	Increase	25
		*M. abscessus-massiliense*	25		
		*M. fortuitum*	6		

## Conclusions

In conclusion, the present study, although not representing a population-based investigation, shows an increase in NTM isolation rate in our setting, which is consistent with the increasing rates seen elsewhere, and provides a snapshot of the prevalent NTM species in our setting. The clinical significance of the increased isolation rate of NTM from human specimens observed in the present study remains largely unknown for the difficulties to interpret whether the NTM isolations are related to colonisation or disease. Further studies involving detailed clinical data are needed to better understand the changes in NTM epidemiology.

## References

[1] Tortoli E (2003). Impact of genotypic studies on mycobacterial taxonomy: the new mycobacteria of the 1990s. Clin Microbiol Rev.

[2] Griffith DE, Aksamit T, Brown-Elliott BA, Catanzaro A, Daley C, Gordin F (2007). An official ATS/IDSA statement: diagnosis, treatment, and prevention of nontuberculous mycobacterial diseases. Am J Respir Crit Care Med.

[3] Tortoli E (2009). Clinical manifestations of nontuberculous mycobacteria infections. Clin Microbiol Infect.

[4] van Ingen J (2013). Diagnosis of nontuberculous mycobacterial infections. Semin Respir Crit Care Med.

[5] Hoefsloot W, van Ingen J, Andrejak C, Angeby K, Bauriaud R, Bemer P (2013). The geographic diversity of nontuberculous mycobacteria isolated from pulmonary samples: an NTM-NET collaborative study. Eur Respir J.

[6] Behr MA, Falkinham JO (2009). Molecular epidemiology of nontuberculous mycobacteria. Future Microbiol.

[7] van der Werf MJ, Ködmön C, Katalinić-Janković V, Kummik T, Soini H, Richter E (2014). Inventory study of non-tuberculous mycobacteria in the European Union. BMC Infect Dis.

[8] Ringshausen FC, Apel RM, Bange FC, de Roux A, Pletz MW, Rademacher J (2013). Burden and trends of hospitalisations associated with pulmonary non-tuberculous mycobacterial infections in Germany, 2005–2011. BMC Infect Dis.

[9] Moore JE, Kruijshaar ME, Ormerod LP, Drobniewski F, Abubakar I (2010). Increasing reports of non-tuberculous mycobacteria in England, Wales and Northern Ireland, 1995–2006. BMC Public Health.

[10] Shin SJ, Lee BS, Koh WJ, Manning EJ, Anklam K, Sreevatsan S (2010). Efficient differentiation of *Mycobacterium avium* complex species and subspecies by use of five-target multiplex PCR. J Clin Microbiol.

[11] Jankovic M, Samarzija M, Sabol I, Jakopovic M, Katalinic Jankovic V (2013). Geographical distribution and clinical relevance of non-tuberculous mycobacteria in Croatia. Int J Tuberc Lung Dis.

[12] Eriksson M, Bennet R, Danielsson N (2001). Non-tuberculous mycobacterial lymphadenitis in healthy children: another lifestyle disease?. Acta Paediatr.

[13] Russell CD, Claxton P, Doig C, Seagar AL, Rayner A, Laurenson IF (2014). Non-tuberculous mycobacteria: a retrospective review of Scottish isolates from 2000 to 2010. Thorax.

[14] van Ingen J, Hoefsloot W, Dekhuijzen PN, Boeree MJ, van Soolingen D (2010). The changing pattern of clinical *Mycobacterium avium* isolation in the Netherlands. Int J Tuberc Lung Dis.

[15] Chien JY, Lai CC, Sheng WH, Yu CJ, Hsueh PR (2014). Pulmonary infection and colonization with nontuberculous mycobacteria, Taiwan, 2000–2012. Emerg Infect Dis.

[16] Marras TK, Mendelson D, Marchand-Austin A, May K, Jamieson FB (2013). Pulmonary nontuberculous mycobacterial disease, Ontario, Canada, 1998–2010. Emerg Infect Dis.

[17] Prevots DR, Shaw PA, Strickland D, Jackson LA, Raebel MA, Blosky MA (2010). Nontuberculous mycobacterial lung disease prevalence at four integrated health care delivery systems. Am J Respir Crit Care Med.

[18] Billinger ME, Olivier KN, Viboud C, de Oca RM, Steiner C, Holland SM (2009). Nontuberculous mycobacteria-associated lung disease in hospitalized persons, United States, 1998–2005. Emerg Infect Dis.

[19] Martín-Casabona N, Bahrmand AR, Bennedsen J, Thomsen VO, Curcio M, Fauville-Dufaux M (2004). Non-tuberculous mycobacteria: patterns of isolation. A multi-country retrospective survey. Int J Tuberc Lung Dis.

[20] Panagiotou M, Papaioannou AI, Kostikas K, Paraskeua M, Velentza E, Kanellopoulou M (2014). The epidemiology of pulmonary nontuberculous mycobacteria: data from a general hospital in Athens, Greece, 2007–2013. Pulm Med.

[21] Henkle E, Hedberg K, Schafer S, Novosad S, Winthrop KL (2015). Population-based incidence of pulmonary nontuberculous mycobacterial disease in Oregon 2007 to 2012. Ann Am Thorac Soc.

[22] Satyanarayana G, Heysell SK, Scully KW, Houpt ER (2011). Mycobacterial infections in a large Virginia hospital, 2001–2009. BMC Infect Dis.

[23] Wu J, Zhang Y, Li J, Lin S, Wang L, Jiang Y (2014). Increase in nontuberculous mycobacteria isolated in Shanghai, China: results from a population-based study. PLoS One.

[24] Kim JK, Rheem I (2013). Identification and distribution of nontuberculous mycobacteria from 2005 to 2011 in cheonan, Korea. Tuberc Respir Dis.

[25] Koh WJ, Chang B, Jeong BH, Jeon K, Kim SY, Lee NY (2013). Increasing recovery of nontuberculous mycobacteria from respiratory specimens over a 10-year period in a tertiary referral hospital in South Korea. Tuberc Respir Dis.

